# Dietary Patterns and Risk of Frailty in Chinese Community-Dwelling Older People in Hong Kong: A Prospective Cohort Study

**DOI:** 10.3390/nu7085326

**Published:** 2015-08-24

**Authors:** Ruth Chan, Jason Leung, Jean Woo

**Affiliations:** 1 Department of Medicine and Therapeutics, The Chinese University of Hong Kong, Shatin, Hong Kong, China; E-Mail: jeanwoowong@cuhk.edu.hk; 2 Jockey Club Centre for Osteoporosis Care and Control, The Chinese University of Hong Kong, Shatin, Hong Kong, China; E-Mail: jason-leung@cuhk.edu.hk

**Keywords:** dietary pattern, frailty, Chinese

## Abstract

Dietary pattern analysis is an emerging approach to investigate the association between diet and frailty. This study examined the association of dietary patterns with frailty in 2724 Chinese community-dwelling men and women aged ≥ 65 years. Baseline dietary data were collected using a food frequency questionnaire between 2001 and 2003. Adherence to *a priori* dietary patterns, including the Diet Quality Index-International (DQI-I) and the Mediterranean Diet Score (MDS) was assessed. Factor analysis identified three *a posteriori* dietary patterns, namely “vegetables-fruits”, “snacks-drinks-milk products”, and “meat-fish”. Incident frailty was defined using the FRAIL scale. Binary logistic regression was applied to examine the associations between dietary patterns and four-year incident frailty. There were 31 (1.1%) incident frailty cases at four years. Every 10-unit increase in DQI-I was associated with 41% reduced risk of frailty in the sex- and age-adjusted model (odds ratio (OR) (95% confidence interval (CI)): 0.59 (0.42–0.85), *p* = 0.004). The association attenuated in the multivariate adjusted model (0.69 (0.47–1.02), *p* = 0.056). No association between other dietary patterns and incident frailty was observed. Our study showed that a better diet quality as characterized by higher DQI-I was associated with lower odds of developing frailty. The contribution of MDS or *a posteriori* dietary patterns to the development of frailty in Chinese older people remains to be explored.

## 1. Introduction

Frailty is considered as a state of multisystem impairments with aging resulting in increased vulnerability to acute stressors [1]. It is often associated with an increased risk of adverse outcomes such as disability, morbidity, dependence, and institutionalization [2,3]. Although the prevalence of frailty varies greatly depending on the frailty measures [3], it is estimated that frailty affects 10.7% of community dwelling people aged 65 and older and its prevalence increases with age. An estimate of 15.7% of older people aged 80 to 84, and 26.1% of those aged 85 and above were classified as frail in the community [4]. With an ageing population in most developed countries, its impact on health and social care costs is substantial and identifying strategies to prevent or treat frailty is therefore of public health importance.

Some evidence suggests that intervention programs incorporating physical, cognitive, social support and nutritional components in early stages of frailty could possibly treat frailty; the optimal intervention for preventing or reversing frailty however remains to be investigated [2,5,6]. Meanwhile, accumulating evidence shows that diet may play a role in preventing or slowing down the onset of frailty. A diet rich in protein, vitamins and antioxidants is suggested to be beneficial for preventing or slowing down this age-related decline in both physical and cognitive functions [7,8,9,10]. However, most previous studies examined the association between diet and frailty using a single nutrient or food group approach and this approach is unlikely to take into account the synergy of various nutrients and food groups in the entire diet [11,12]. Therefore, dietary pattern analysis has been applied as an alternative approach to relate diet to frailty status.

To our knowledge, only few observational studies [13,14,15,16] have been conducted to examine the association between dietary patterns and frailty. All these studies were however conducted in Caucasian populations. In view of the scarcity of evidence on this topic and the fact that Chinese diets are different from those of Caucasian population, the present study aimed to examine the relationship of *a priori* and *a posteriori* diet patterns with incident frailty in Chinese community-dwelling older people in Hong Kong.

## 2. Experimental Section

### 2.1. Study Population

A total of 2000 Chinese men and 2000 Chinese women aged 65 years or over living in the community in Hong Kong were recruited on voluntary basis to participate in this study between August 2001 and December 2003. Details of this prospective cohort study have been described previously [17]. In brief, participants were able to walk or take public transport to the study site. They were recruited using a stratified sampling method and there were approximately 33% of each of these age groups: 65–69, 70–74, and 75+. Participants attended the four-year follow-up between August 2005 and November 2007. Mean (standard error (SD)) follow-up year was 3.9 (0.1) years. This study was conducted in compliance with the Declaration of Helsinki and was approved by the Clinical Research Ethics Committee of the Chinese University of Hong Kong. All participants gave written informed consent. Participants who were pre-frail or frail at baseline as defined using the FRAIL scale [18], had incomplete or invalid dietary or demographic data, and discontinued the four-year follow-up, were excluded from the prospective incident analysis. The sample size for the final analysis was 2724.

### 2.2. Questionnaire and Anthropometric Measurements

A standardized interview was conducted to capture information on demographics, lifestyle and previous health. Information regarding the duration and level of previous and current use of cigarettes, cigars and pipes was obtained. Smoking status was divided into former smoking (at least 100 cigarettes smoked in a lifetime), current smoking or never smoking. Alcohol use was asked and drinking status was defined as never, former or current. Current drinkers referred to those who drank ≥12 drinks of beer, wine (including Chinese wine) or liquor over the past year. Medical history was obtained at baseline based on participants’ self-report of their physician’s diagnoses, supplemented by the identification of drugs brought to the interviewers. Depressive symptoms were assessed using the Geriatric Depression Scale (GDS) [19] with a score of 8 or above representing depressive symptoms, validated in elderly Chinese subjects [20]. Cognitive function was evaluated using the Cognitive Screening Instrument for Dementia (CSID) with a cutoff value for probable or borderline dementia of 29.5 or below [21]. Physical activity level was assessed using the Physical Activity Scale of the Elderly (PASE) [22]. Higher score indicates higher physical activity level. Information regarding any difficulty in performing activities of daily living, such as walking two to three blocks outside on level ground and climbing 10 steps without resting were also obtained. Body weight was measured with participants wearing a light gown, using the Physician Balance Beam Scale (Healthometer, Illinois, USA). Height was measured with the Holtain Harpenden stadiometer (Holtain Ltd., Crosswell, UK). Body mass index (BMI) was calculated as body weight in kg/(height in m)^2^.

### 2.3. Dietary Assessment

Dietary intake was assessed at baseline using a validated semi-quantitative food frequency questionnaire (FFQ) [23]. Details have been reported previously [24]. Trained research staff asked each participant to report the frequency and the usual amount of consumption of each food item over the past year. Portion size was quantified using a catalogue of pictures of individual food portions. Daily amount of consumption of major food groups including cereals, egg and egg products, fish and shellfish, fruits and dried fruits, legumes/nuts/seeds, meat and poultry, milk and milk products, and vegetables was calculated. Mean daily nutrient intake was calculated using food tables derived from McCance and Widdowson [25] and the Chinese Medical Sciences Institute [26].

### 2.4. A Priori and A Posteriori Dietary Pattern Scores

The Dietary Quality index-International (DQI-I) was generated using the method described by Kim *et al.* [27] and details have been described elsewhere [28]. In brief, four major aspects of the diet are assessed in the index, namely variety, adequacy, moderation and overall balance. The DQI-I total score ranges from 0 to 94 with higher score indicating better diet quality. A Mediterranean Diet Score (MDS) was used to assess the adherence to the Mediterranean diet and it was calculated using the revised method described by Trichopoulou *et al.* [29]. Details of its calculation have been reported previously [30]. The total MDS ranges from 0 (minimal adherence) to 9 (maximal adherence).

Details of dietary pattern scores derived by the factor analysis have been described elsewhere [24]. In brief, each food item in the FFQ were aggregated into 32 food groups according to the similarity of food type and nutrient composition. The food groups were then energy adjusted, in which the energy intake from each food group was divided by total energy intake and multiplied by 100, and were expressed as percentage contribution to total energy [31]. Factor analysis was performed with varimax rotation using the 32 food groups [32]. Factors were retained based on an eigenvalues greater than 1.0, a scree plot as well as the interpretability [33]. The factor score for each pattern were then calculated for each participant through summing intakes of food items weighted by their factor loadings. A higher score represented greater conformity with the derived pattern. Three dietary patterns were identified in the present study, namely “vegetables-fruits”, “snacks-drinks-milk products” and “meat-fish” (Table 1) [24].

**Table 1:** Food group factor loading ^a^ for three dietary patterns.

Food Groups	Dietary Patterns		
	Factor 1: Vegetables-Fruits	Factor 2: Snacks-Drinks-Milk Products	Factor 3: Meat-Fish
Other vegetables	**0.58**	−0.06	0.02
Tomatoes	**0.49**	0.03	−0.01
Dark green and leafy vegetables	**0.43**	−0.26	−0.02
Cruciferous vegetables	**0.43**	−0.05	−0.06
Starchy vegetables	**0.42**	0.03	0.00
Soy	**0.42**	0.08	0.11
Fruits	**0.40**	0.03	−0.01
Legumes	**0.34**	−0.01	0.02
Mushroom and fungi	**0.22**	0.06	−0.07
Fats and oils	**−0.37**	−0.21	0.15
Condiments	−0.05	**0.48**	−0.13
Coffee	−0.15	**0.42**	−0.17
Fast food	−0.03	**0.37**	0.04
Nuts	0.12	**0.37**	−0.03
French fries and potato chips	−0.03	**0.37**	0.09
Milk and milk products	0.08	**0.31**	−0.14
Whole grains	0.14	**0.30**	−0.17
Sweets and desserts	0.02	**0.29**	0.08
Beverages	−0.03	**0.22**	0.09
Dim sum	−0.17	−0.11	**0.56**
Red and processed meats	−0.07	0.06	**0.46**
Poultry	0.06	0.11	**0.45**
Fish and seafood	0.22	−0.17	**0.37**
Wine	−0.14	0.10	**0.20**
Refined grains	−0.25	−0.50	**−0.69**
Cakes, cookies, pies and biscuits	0.06	0.14	0.19
Eggs	0.07	0.19	0.05
Organ meats	−0.08	0.15	0.12
Others	0.01	0.07	0.03
Preserved vegetables	−0.02	0.08	0.00
Soups	0.00	0.00	−0.01
Tea	0.00	0.15	0.03
% variance explained	6.2	5.4	5.1

^a^ Factor loadings with absolute value ≥0.2 are shown in bold. For food group loads more than one dietary pattern, only the highest absolute value of loading is bolded.

### 2.5. Definition of Frailty

The five-item FRAIL scale proposed by Morley *et al.* was used to assess frailty status [18]. The FRAIL scale is comparable with other existing short screening tools and tools based on the multiple-deficits model in predicting mortality and physical limitations [3]. A score of 1 is assigned to each of the five components: fatigue, resistance (*i.e.*, inability to climb one flight of stairs), ambulation (*i.e.*, inability to walk one block), having more than five diseases, and weight loss of more than 5%. The equivalent variables used for construction of this score from the database in the present study are reporting no energy, inability to climb up 10 steps, unable to walk two to three blocks, more than five diseases, and BMI below 18.5 kg/m^2^. Frailty scores range from 0 to 5 and represent frail (3 to 5), pre-frail (1 to 2), and robust (0) health status.

### 2.6. Statistical Analysis

Statistical analyses were performed using the statistical package SPSS version 21.0 (SPSS Inc., Chicago, IL, USA). Data was checked for normality using descriptive analysis. Independent student’s *t* test and chi square test or Fisher exact test were used to examine the baseline differences in mean age, BMI, energy intake, dietary pattern scores, and PASE, and also the differences in the distribution of sex, education level, smoking habit, alcohol use, living arrangement, marital status, GDS category and CSID category between participants included and participants excluded for data analysis, and between those who were frail and those who were not at the four-year follow-up. Pearson’s and Spearman’s rank correlations were used to examine the correlation between each dietary pattern score and various nutrient and food group intakes whenever appropriate.

The association between each diet pattern score and the risk of being frail was analyzed using logistic regression models. The first model was the unadjusted model and the second model was adjusted for sex and age (continuous). The third model was further adjusted for baseline BMI (continuous), daily energy intake (continuous), PASE (continuous), education level (primary or below *vs.* secondary or above), current smoker status (yes *vs.* no), current alcohol status (yes *vs.* no), GDS category (<8 *vs.* ≥8), CSID category (≤29.5 *vs.* >29.5), living alone (yes *vs.* no) and marital status (married/cohabited *vs.* widowed/separated/divorced/single/never married). All tests were 2-sided and *p* values less than 0.05 were considered statistically significant.

## 3. Results

Excluded participants were older and less physically active, had lower BMI, lower energy intake and diet quality, and lower education attainment in comparison to included participants (*p* < 0.05). Excluded participants were also more likely to be living alone and to be a current smoker, and to have depressive symptoms, cognitive impairment and worse marital status than those who were included in the analysis (*p* < 0.05) (details not shown).

A total of 31 (1.1%) cases were newly identified as frail at the four-year follow-up. Baseline characteristics of participants with frailty and participants without frailty at four years are shown in Table 2. Frail participants were older and physically less active, and had lower energy intake, DQI-I and “snacks-drinks-milk products” score than non frail participants. They also had lower education attainment and were more likely to have worse marital, cognitive and depressive conditions than participants without frailty.

**Table 2:** Baseline characteristics of participants by four-year frailty status (*n* = 2724).

	Total (*n* = 2724)	Non frail ^a^ (*n* = 2693)	Frail ^a^ (*n* = 31)	*p*-Value ^b^
Age (year)	71.8 (4.8)	71.8 (4.7)	76.0 (5.8)	<0.001
BMI (kg/m^2^)	24.0 (2.9)	24.0 (2.9)	24.2 (4.1)	0.798
PASE	95.0 (43.5)	95.2 (43.5)	81.3 (45.0)	0.076
Energy intake (kcal/day)	1854.1 (571.8)	1857.5 (572.4)	1562.1 (428.3)	0.001
Female (%)	50.3	50.1	64.5	0.110
Education (%)				
Primary or below	69.9	69.7	87.1	0.036
Secondary or above	30.1	30.3	12.9	
GDS ≥ 8 (%)	7.0	6.8	25.8	0.001
CSID ≤ 29.5 (%)	23.6	23.4	38.7	0.046
Living alone (%)	9.9	9.8	19.4	0.119
Marital status (%)				
Married	72.9	73.2	48.4	0.002
Others ^c^	27.1	26.8	51.6	
Current alcohol use (%)	13.8	13.9	6.5	0.302
Current smoker (%)	5.6	5.6	9.7	0.251
DQI-I	65.1 (9.2)	65.1 (9.1)	60.5 (12.1)	0.041
MDS	4.1 (1.5)	4.1 (1.5)	4.0 (1.4)	0.746
Factor 1: Vegetables-fruits	0.03 (0.97)	0.03 (0.97)	−0.22 (1.12)	0.156
Factor 2: Snacks-drinks-milk products	0.04 (1.02)	0.04 (1.03)	−0.39 (0.73)	0.003
Factor 3: Meat-fish	−0.02 (0.99)	−0.02 (0.99)	−0.17 (0.98)	0.411

^a^ Frailty score (0–5) was defined using the five-item FRAIL scale [18]. A score of ≥3 was classified as frail; ^b^ *p-*value between non-frail participants and frail participants by independent Student’s *t-*test for continuous variables and chi square test or Fisher exact test for categorical variables; ^c^ Widowed, separated, divorced, single or never married; BMI: body mass index; PASE: Physical Activity Scale of the Elderly; GDS: Geriatric Depression Scale; CSID: Cognitive Screening Instrument for Dementia; DQI-I: Diet Quality Index-International; MDS: Mediterranean Diet Score.

DQI-I, MDS and “vegetables-fruits” dietary pattern scores showed similar correlations with various nutrients. These three scores were positively associated with the intakes of protein, fiber, vitamins C and D, and minerals. Although “snacks-drinks-milk products” dietary pattern scores were also positively associated with fiber, vitamin C, magnesium and potassium, the associations were weaker than those of DQI-I, MDS and “vegetables-fruits” dietary pattern scores. While “snacks-drinks-milk products” dietary pattern scores and “meat-fish” dietary pattern scores were both positively associated with protein intake, the associations of other nutrients, such as fiber, calcium and vitamin D were far weaker in the “meat-fish” dietary pattern as compared to the “snacks-drinks-milk products” pattern. Overall DQI-I, MDS and “vegetables-fruits” dietary pattern scores were positively associated with intakes of plant-based foods, egg and egg products, as well as fish and shellfish, and inversely associated with meat and poultry consumption. Similarly, a positive correlation was observed for the “snacks-drinks-milk products” dietary pattern score and the “meat-fish” dietary pattern score with the intakes of plant based foods. However, the magnitude of the correlations was smaller in comparison to DQI-I, MDS and “vegetables-fruits” dietary pattern scores (Table 3).

Participants with a higher DQI-I score had a lower risk of being frail in both the crude and the sex- and age-adjusted models (*p* < 0.05). These associations attenuated when the model was further adjusted for other demographic and lifestyle factors (Table 4). Similarly, the significant inverse association between the “snacks-drinks-milk products” score and the risk of frailty disappeared after controlling for other demographic and lifestyle factors. There was no association of MDS, “vegetables-fruits” pattern or “meat-fish” pattern with incident frailty. Sensitivity analysis after excluding those with various chronic diseases including diabetes, hypertension, cardiovascular disease and stroke at baseline or those with cognitive impairment defined using CSID ≤ 29.5 at baseline showed similar results (Table 4).

**Table 3:** Correlation between each dietary pattern score and selected nutrient intakes and food groups (*n* = 2724).

Nutrient/Food Group	DQI-I	*p*-Value	MDS	*p*-Value	Factor 1: Vegetables-Fruits	*p*-Value	Factor 2: Snacks-Drinks-Milk Products	*p* value	Factor 3: Meat-Fish	*p*-Value
	*r*		*r*		*r*		*r*		*r*	
Energy (kcal/day)	0.163	<0.001	0.246	<0.001	−0.047	0.014	0.293	<0.001	0.197	<0.001
Protein (g/day) ^a^	0.241	<0.001	0.217	<0.001	0.134	<0.001	0.344	<0.001	0.356	<0.001
Protein (% energy/day) ^a^	0.160	<0.001	0.051	0.008	0.317	<0.001	0.232	<0.001	0.388	<0.001
Fiber (g/day) ^a^	0.579	<0.001	0.447	<0.001	0.573	<0.001	0.188	<0.001	−0.002	0.902
Calcium (mg/day) ^a^	0.492	<0.001	0.281	<0.001	0.364	<0.001	0.344	<0.001	−0.015	0.449
Magnesium (mg/day) ^a^	0.493	<0.001	0.412	<0.001	0.350	<0.001	0.078	<0.001	−0.038	0.047
Potassium (mg/day) ^a^	0.286	<0.001	0.288	<0.001	0.239	<0.001	0.175	<0.001	0.308	<0.001
Vitamin C (mg/day) ^a^	0.544	<0.001	0.386	<0.001	0.517	<0.001	0.042	0.027	0.084	<0.001
Vitamin D (IU/day) ^a^	0.030	0.118	0.012	0.517	0.076	<0.001	0.299	<0.001	0.040	0.038
Cereals (g/day) ^b^	0.325	<0.001	0.331	<0.001	−0.168	<0.001	−0.103	<0.001	−0.325	<0.001
Egg and egg products (g/day) ^b^	0.084	<0.001	0.054	0.005	0.140	<0.001	0.282	<0.001	0.125	<0.001
Fast food (g/day) ^b^	−0.004	0.983	−0.052	0.007	−0.019	0.318	0.433	<0.001	0.078	<0.001
Fish and shellfish (g/day) ^b^	0.153	<0.001	0.377	<0.001	0.210	<0.001	−0.011	0.555	0.409	<0.001
Fruits and dried fruits (g/day) ^b^	0.435	<0.001	0.379	<0.001	0.349	<0.001	0.157	<0.001	0.129	<0.001
Legumes, seeds and nuts (g/day) ^b^	0.301	<0.001	0.429	<0.001	0.371	<0.001	0.215	<0.001	0.118	<0.001
Meat and poultry (g/day) ^b^	−0.075	<0.001	−0.119	<0.001	−0.042	0.030	0.252	<0.001	0.576	<0.001
Milk and milk products (g/day) ^b^	0.113	<0.001	−0.261	<0.001	0.072	<0.001	0.445	<0.001	−0.027	0.152
Vegetables (g/day) ^b^	0.438	<0.001	0.485	<0.001	0.665	<0.001	0.024	0.216	0.126	<0.001

^a^ Nutrients with logarithmic transformation; ^b^ By Spearman’s rank correlation.

**Table 4:** Crude and adjusted associations between each dietary pattern score and incident frailty (*n* = 2724).

Dietary Pattern Score	Unadjusted OR	95% CI	*p*-Value	Age and Sex Adjusted	95% CI	*p* value	Multivariate Adjusted ^a^	95% CI	*p*-Value
				OR			OR		
All 2724 subjects									
DQI-I (per 10-unit increase)	0.61	0.43–0.86	**0.005**	0.59	0.42–0.85	**0.004**	0.69	0.47–1.02	0.056
MDS	0.96	0.77–1.21	0.746	0.97	0.77–1.23	0.815	1.06	0.83–1.36	0.638
Factor 1: Vegetables-fruits	0.73	0.48–1.12	0.151	0.67	0.43–1.04	0.073	0.76	0.48–1.21	0.249
Factor 2: Snacks-drinks-milk products	0.58	0.36–0.91	**0.018**	0.64	0.40–1.02	0.062	0.78	0.48–1.28	0.331
Factor 3: Meat-fish	0.86	0.59–1.24	0.411	0.89	0.61–1.30	0.542	0.95	0.63–1.41	0.787
1255 subjects without selected chronic diseases at baseline									
DQI-I (per 10-unit increase)	0.37	0.20–0.68	**0.001**	0.36	0.20–0.66	**0.001**	0.41	0.20–0.81	**0.009**
MDS	1.04	0.69–1.56	0.858	1.04	0.69–1.57	0.858	1.29	0.81–2.08	0.286
Factor 1: Vegetables-fruits	0.50	0.22–1.18	0.114	0.48	0.20–1.18	0.108	0.55	0.22–1.37	0.197
Factor 2: Snacks-drinks-milk products	0.45	0.19–1.06	0.069	0.48	0.21–1.14	0.095	0.45	0.18–1.15	0.093
Factor 3: Meat-fish	1.11	0.61–2.01	0.729	1.15	0.63–2.11	0.656	1.24	0.64–2.43	0.523
2082 subjects without cognitive impairment at baseline ^b^									
DQI-I (per 10-unit increase)	0.55	0.35–0.87	**0.010**	0.53	0.34–0.83	**0.006**	0.59	0.36–0.96	**0.033**
MDS	0.86	0.64–1.15	0.302	0.86	0.64–1.16	0.330	0.93	0.68–1.28	0.669
Factor 1: Vegetables-fruits	0.69	0.40–1.20	0.187	0.60	0.34–1.06	0.076	0.64	0.36–1.15	0.135
Factor 2: Snacks-drinks-milk products	0.49	0.27–0.90	**0.022**	0.52	0.29–0.96	**0.037**	0.59	0.31–1.12	0.106
Factor 3: Meat-fish	0.87	0.55–1.39	0.556	0.91	0.56–1.46	0.691	0.96	0.58–1.59	0.879

^a^ Further adjusted for body mass index (BMI), energy intake, Physical Activity Scale of the Elderly (PASE), education level, smoking status, alcohol use, Geriatric Depression Scale (GDS) category, Cognitive Screening Instrument for Dementia (CSID) category, living alone and marital status at baseline; ^b^ CSID category was not included in the multivariate adjusted model; DQI-I: Diet Quality Index-International; MDS: Mediterranean Diet Score; OR: odds ratio; CI: 95% confidence interval.

## 4. Discussion

Our study revealed an inverse association between DQI-I and incident frailty in Chinese community-dwelling older people. A better diet quality as characterized by a higher DQI-I was associated with a lower odds of developing frailty over four-year follow-up, suggesting that a better diet quality may be beneficial for lowering the risk of being frail in older people. Sensitivity analysis by excluding participants with chronic diseases or cognitive impairment from the analysis further showed that a 10-unit increase in DQI-I was associated with around 41% to 59% of reduced risk of being frail. However, no association between MDS or *a posteriori* dietary pattern scores and incident frailty was observed in the present study.

To our knowledge, only few studies have been made to examine the relationship between diet and frailty using a dietary pattern analysis approach. Our observations that a higher DQI-I was associated with a lower risk of frailty were consistent with those reported in a sample of community-dwelling older men participating in the Osteoporotic Fractures in Men (MrOS) in the US. In the MrOs US study, macronutrient intake showed no associations with frailty status whereas overall diet quality as measured using the Diet Quality Index Revised (DQI-R) was consistently associated with both prevalent and incident frailty status in this cohort [13]. In contrast, the findings that no association between MDS and frailty status in our study were different from those reported in previous cross-sectional [15] and prospective studies [16]. In a group of 192 community-dwelling older volunteers living in Germany, a higher adherence to a Mediterranean Diet Score was associated with a reduced likelihood of being frail and in particular of the frailty criteria “low walking speed” and “low physical activity” [15]. Findings of a prospective study among 690 older people living in the community also suggested that a higher adherence to a Mediterranean-style diet was associated with a lower risk of development of frailty over six years of follow-up [16]. Several reasons may explain the inconsistent results between our study and these previous studies. Although the Chinese diet is similar to the Mediterranean diet in that both are characterized by high vegetable and fruit consumption and low meat consumption, the consumption of legumes, nuts, olive oil and wine was lower in the Chinese diet in comparison to the traditional Mediterranean diet. Second, the differences in the study design between our study and the previous studies, including the use of different frailty measures and the inclusion of different covariates in the adjusted models may partly account for the inconsistent results between our study and the previous studies.

In this study, none of the *a posteriori* dietary patterns was associated with incident frailty. The results were somewhat unexpected. To our knowledge, only one study has examined the association of *a posteriori* dietary patterns and frailty. Using factor analysis, León-Muñoz and colleagues identified a “prudent” pattern and a “Westernized” pattern in 1872 non-institutionalized older adults. Higher adherence to the “prudent” pattern as characterized by high intake of olive oil and vegetables showed an inverse dose-response relationship with frailty at 3.5 years of follow-up. In contrast, higher adherence to the “Westernized” pattern as characterized by high intake of refined cereals, whole dairy, and red and processed meat, was associated with an increased risk of slow walking speed and weight loss [14]. In our analysis, a non-significant inverse trend was consistently observed between both the “vegetables-fruits” pattern score and the “snacks-drinks-milk products” pattern score and incident frailty. The small number of cases with incident frailty in our study may limit the ability to detect any significant association between these dietary patterns and frailty. Among all *a priori and a posteriori* dietary patterns examined in the present study, only the DQI-I showed significant associations with frailty status. From the correlations of nutrients and food groups with all these dietary pattern scores, DQI-I is more likely to represent a balanced diet in terms of energy and nutrient intakes as well as various food group consumption in comparison to other diet patterns. These findings may imply that a diet of adequate energy intake, optimal protein intake, reduced consumption of fast food, and being rich in plant-based and antioxidant containing foods, such as vegetables and fruits is important for delaying the onset of frailty in older adults. Although the exact biological mechanism that causes frailty in older adults are still poorly understood, such dietary patterns appear beneficial for frailty prevention possibly via its effects on reducing oxidative stress and inflammation that occurs with ageing [34] and on modulating peripheral risk factors such as cardiovascular diseases, metabolic syndrome, muscle protein synthesis and weight status [7,11,35]. To more effectively prevent or tackle the frailty problem, the implementation of frailty screening by health care professionals in older persons, the prescription of vitamin D supplements in frail persons who are vitamin D deficient, as well as the provision of exercise and nutritional interventions against physical frailty have also been recommended by an international consensus group [5,36].

The strengths of the present study are its prospective design and inclusion of a wide range of potential demographic and lifestyle factors in the analysis. However, there are several limitations in our study. First, dietary data were collected using a FFQ and this may be subject to recall bias. Factors, such as age-related decline in cognitive function and memory may affect the validity of self-reported dietary information. Misreporting of energy intake, in particular underreporting is also common in elderly. These factors are considered as inherent problems with dietary assessment among elderly population [37,38,39] and the measurement errors may attenuate associations between diet patterns and frailty outcome. However, we tried to exclude subjects with cognitive impairment at baseline in the sensitivity analysis and the significant association between DQI-I and incident frailty remained. These observations further assure a potential link between DQI-I and frailty in our studied population. Second, although we controlled for various factors in our analysis, residual confounding from other factors in relationship to frailty, such as vitamin D status as suggested by others [40] might still be present. Previous findings based on the same Chinese male cohort however suggest that vitamin D deficiency was uncommon and vitamin D status might not play an important role in physical performance measures or muscle mass in Chinese community-dwelling older men in Hong Kong [41]. Third, our sample was in general of a higher education attainment in comparison to the general Hong Kong population. They were also volunteers who were able to walk or take public transport to the study site, thus our participants were likely to be more health conscious and have good baseline physical functions. Therefore, the results may not be generalized to the general population. Besides, the *a* *posteriori* dietary pattern approach is dependent on the sample information, the pattern derived is therefore highly specific to the diet of the studied population. The results or patterns generated from our study may not be generalized to the general target population or applicable to other countries especially with different cultures and dietary practices.

## 5. Conclusions

Our study showed that a better diet quality as characterized by DQI-I was associated with lower risk of frailty in Chinese community-dwelling older people. Since DQI-I is more likely to represent a balanced diet in terms of energy and nutrient intakes as well as various food group consumption, our findings may imply that a diet of adequate energy intake, optimal protein intake, reduced consumption of fast food, and being rich in plant-based and antioxidant containing foods, such as vegetables and fruits is important for delaying the onset of frailty in Chinese older adults. The contribution of MDS or *a posteriori* dietary patterns to the development of frailty in Chinese older people remains to be explored.
